# Research progress on damage-associated molecular patterns in acute kidney injury

**DOI:** 10.3389/fimmu.2025.1590822

**Published:** 2025-07-10

**Authors:** Jiajia Li, Zhangxue Hu

**Affiliations:** Department of Nephrology, West China Hospital, Sichuan University, Chengdu, China

**Keywords:** AKI, DAMPs, PRRs, inflammation, immunity

## Abstract

Acute kidney injury (AKI) is a clinical syndrome characterized by a sudden dysfunction of the kidney, which is common worldwide, with a relatively high incidence and mortality rate. Damage to the proximal renal tubule is a pathological hallmark of AKI, and inflammation triggered by the overactivation of the immune system is a common cause of proximal renal tubular injury, which is an important contributing factor in AKI exacerbation. Damage-associated molecular patterns (DAMPs) are endogenous molecules released by cells in response to external stimuli that can trigger an inflammatory response by binding to specific pattern recognition receptors (PRRs). Numerous studies have indicated that when the kidney is exposed to external stress or chemical stimuli, injured cells actively secrete or passively release various DAMPs, which can exacerbate or attenuate kidney injury by stimulating or inhibiting the inflammatory response through binding to the appropriate receptor. Currently, there is a lack of early diagnostic biomarkers and specific therapeutic strategies for AKI in the clinic have been established, and given the important role of the release of DAMPs in the regulation of inflammatory response, they will highly likely become favorable candidate biomarkers and clinical therapeutic targets for AKI. Therefore, a deeper understanding of the types of DAMPs and the specific mechanisms of their actions will provide more possibilities for the specific AKI diagnosis and treatment.

## 1 Introduction

Acute kidney injury (AKI) is a clinical syndrome of rapid decline in renal function caused by various etiologies (1, 2). The decrease in the estimated glomerular filtration rate (eGFR) causes fluid retention and acid–base imbalance, leading to injuries of multiple systems such as the heart, brain, lung, and gastrointestinal (3–6). Severe AKI may cause chronic kidney disease (CKD) and even permanent loss of kidney function. Given its high incidence, mortality and risk for CKD, AKI has remained a global health problem (7–10). The incidence of AKI in patients during hospitalization was reported approximately 10%-15% (11). However, among patients in the intensive care unit (ICU), the incidence became as high as 50% (12). A Canadian study that included approximately 200,000 patients hospitalized for AKI revealed that the 1-year mortality rate for patients with AKI was approximately 28%. Among those who survived, approximately 14% were rehospitalized due to recurrence, with approximately 45% dying within 1 year of hospitalization (13). In China, the all-cause hospital mortality rate of patients with AKI reached 12.4%, and most of them died of multiple organ dysfunction and sepsis (14). Furthermore, some cases of AKI progress to CKD, even end-stage renal disease, with an unfavorable prognosis (5, 15). Apart from the above characteristics, the high hospitalization rate of patients with AKI and the large proportion of medical costs should not be ignored. According to a nationwide cross-sectional study conducted in China, approximately 1.4 million people with AKI were hospitalized in 2013, with hospitalization costs amounting to approximately $13 billion, accounting for 10% of China’s total healthcare expenditure (14). Despite the high proportion of AKI-related mortality and medical costs, no specific drugs have been approved for the treatment of patients with AKI. Current clinical management includes addressing the underlying causes, controlling blood glucose and blood pressure, avoiding nephrotoxic drugs, volume management, hemodynamic monitoring, and renal replacement therapy. Though these approaches partially improve the prognosis of patients with AKI, the CKD risk and death in patients with AKI remains dismal, and new therapeutic strategies need to be explored (4, 16–18).

Factors commonly associated with AKI development include ischemia–reperfusion injury (IRI), sepsis, hemodynamic alterations, systemic inflammation, and use of nephrotoxic drugs, which are often associated with sterile inflammation (3, 6, 10). In the early stage of AKI, various immune cells are recruited to the kidney, releasing proinflammatory mediators and exacerbating renal impairment. In the later AKI stage, the dynamic interaction of pro-inflammatory and anti-inflammatory mediators released by immune cells mediates AKI development, ultimately causing the inflammation to subside and renal tissue damage to be repaired or progressing to CKD (19–21). Therefore, an in-depth study of the mechanisms that regulate sterile renal inflammation will help in AKI prevention and treatment.

Sterile inflammation is an inflammatory response that occurs in the absence of pathogens and usually relies on the release of damage-associated molecular patterns (DAMPs) (22, 23). DAMPs are endogenous molecules released by cells in response to unfavorable stimuli from the internal or external environment. These endogenous molecules are normally sequestered in their respective intracellular compartments under physiological conditions (24, 25). However, when cells are exposed to deleterious stimuli, they are released into the extracellular space, activating the immune system and triggering a sterile inflammatory response (26–28). DAMPs are mainly released passively by dying cells, and the former includes various forms of death such as necrosis, necroptosis, and pyroptosis, which lead to DAMPs leakage through the destruction of the plasma membrane or formation of channel pores into the extracellular space (28, 29). In addition to passive release, some DAMPs can be actively secreted from living cells through vesicular transport by docking and fusion with the plasma membrane through the exosomal and lysosomal exocytosis pathways (30, 31). These released DAMPs bind to specific pattern recognition receptors (PRRs), such as toll-like receptors (TLRs) and receptor for advanced glycation end products (RAGE), inducing nuclear factor-kappa B (NF-κB) transcription and the activation of the NOD-like receptor family pyrin domain-containing protein 3 (NLRP3) inflammasome, which promotes the recruitment of various immune cells and the release of inflammatory factors, which subsequently trigger a sterile inflammatory response (22, 25, 28, 32).

**Figure 1:**
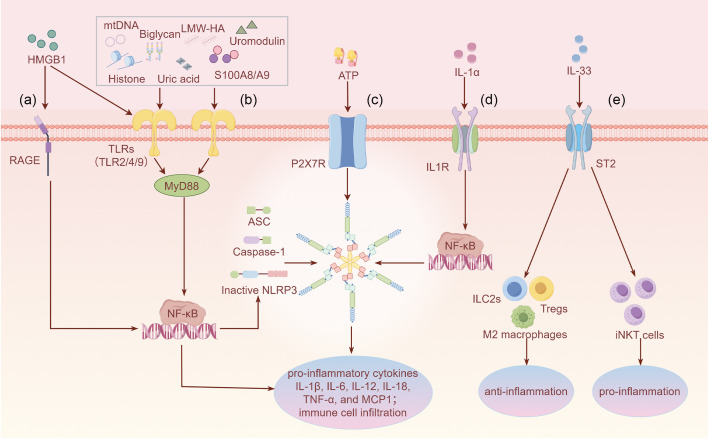
Overview of the specific functions of DAMPs in AKI through binding to different PRRs **(a)** HMGB1 activates RAGE, leading to downstream NF-κB signaling and inflammatory factor release. **(b)** Most DAMPs such as HMGB1, histones, uric acid, mtDNA, uromodulin, biglycan, LMW-HA, and S100A8/A9 activate TLR2/4/9, exacerbating the inflammatory signaling cascade. Only the downstream canonical MyD88/NF-κB signaling pathway is shown. **(c)** ATP regulates NLRP3 inflammasome signaling through the activation of P2X7R and subsequently promotes inflammatory responses. **(d)** IL-1α activates IL-1R, promoting NF-κB translocation to the nucleus and activating inflammatory signaling. **(e)** IL-33 activates ST2, which promotes polarization of M2 macrophages and induces the expansion of ILC2s and Tregs to fight inflammation. Meanwhile, IL-33 binds to ST2 on the surface of iNKT cells, amplifying inflammatory injury. IL-33 exhibits dual functions, which may depend on the specific immune microenvironment. Footnote: **Figure 1** only shows the function of IL-1α and IL-33 as DAMPs rather their function as secreted cytokines.

The death of proximal renal tubular epithelial cells is a key factor that leads to AKI, and oxidative stress, necrosis, apoptosis, and inflammation are crucial in AKI development (20, 33–35). Given that inflammation is an important AKI driver and DAMPs are crucial in regulating inflammation, this review focuses on the role of the inflammatory response participated by DAMPs in AKI. When the kidney suffers from adverse stimuli such as major surgical trauma or nephrotoxic drugs, inflammatory DAMPs released by damaged cells participate in AKI development by binding to the PRRs, stimulating and amplifying inflammatory response signals (36–38). Interestingly, not all DAMPs exacerbate AKI, and some DAMPs have anti-inflammatory effects that promote the repair of damaged tissues and protect the kidney (39–41). Therefore, an in-depth understanding of certain roles played by different DAMPs during the immune response and damage–repair process involved in AKI may contribute to the early diagnosis and discovery of therapeutic targets for AKI. This review focuses on the types of DAMPs in AKI (**Table 1**) and describes the functions of DAMPs in AKI via binding to different PRRs (**Figure 1**).

**Table 1:** Overview of the types of DAMPs in AKI.

DAMPs	Receptor	Inflammation response
HMGB1 (oxidized form)	RAGE, TLR2, TLR4	context-dependent proinflammation
Histones	TLR2, TLR4, TLR9	proinflammation
IL-1α	IL-1R	proinflammation
IL-33	ST2	context-dependent pro- or anti-inflammation
Uric acid	TLR4	proinflammation
ATP	P2X7R	context-dependent proinflammation
mtDNA	TLR9	proinflammation
Uromodulin	TLR4	proinflammation
Biglycan	TLR2, TLR4	proinflammation
LMW- HA	TLR2, TLR4	proinflammation
S100A8/A9	TLR4	proinflammation

## 2 Functions and release mechanisms of DAMPs

### 2.1 High mobility group box 1

HMGB1 is a non-histone DNA-binding protein that is widely expressed in the cellular environment and plays a crucial role in regulating DNA replication, DNA repair, and nucleosome stability, which are essential for cell growth and differentiation (42, 43). In response to adverse external stimuli, HMGB1 can be released passively by necrotic cells because of plasma membrane destruction (44, 45). Cells undergoing necroptosis, a regulated form of necrotic cell death, involving specific signaling molecules such as receptor-interacting protein kinase 1 and 3, and mixed-lineage kinase domain-like protein, also release HMGB1, which ultimately leads to membrane depolarization and cell rupture (46–49). In addition, HMGB1 can be actively secreted by immune cells, which involves multiple post-translational modifications (PTMs) of HMGB1, including oxidation, acetylation, lactation, and phosphorylation. Various PTMs promote interactions between HMGB1 and the nuclear transport receptors that mediate the translocation of HMGB1 from the nucleus to the cytoplasm and its subsequent release into the extracellular space through the exosomal and lysosomal exocytosis pathways (43, 50–52). Notably, the proinflammatory function of HMGB1 is determined by its redox status, and the oxidized form of HMGB1, but not its reduced form, determines the proinflammatory activity of HMGB1 once it is released (53). The oxidized form of extracellular HMGB1 interacts with TLR2, TLR4, and RAGE, expressed on various immune cells, including monocytes, macrophages, and dendritic cells, activating NF-κB transcription and inducing the expression of proinflammatory cytokines, such as interleukin-6 (IL-6), tumor necrosis factor α (TNF-α), and monocyte chemoattractant protein 1 (MCP1), leading to inflammation and worsening injury of the kidney (54–57). Several clinical studies have shown the high serum level of HMGB1 in patients suffering from AKI (58, 59). Studies have reported that HMGB1 inhibitors (60–62) and HMGB1-neutralizing antibodies (63, 64) are effective in improving kidney function and attenuating kidney damage in various AKI mouse models.

### 2.2 Histones

Histones are basic proteins with highly conserved sequences found in the nucleus and influence the stabilization chromatin structure and regulate gene expression under normal physiological conditions (65, 66). Histones can be released passively during apoptosis and necrosis, triggering an inflammatory response by binding to the corresponding PRRs (67–70). Recently, neutrophil extracellular traps (NETs) are a widely reported form of histone release, an extracellular DNA mesh-like structure composed of histones and anti-microbial protein, which are generally released by dying neutrophils (71). Adverse stimuli induce peptidylarginine deiminase 4 (PAD4) and neutrophil elastase (NE) to initiate chromatin decondensation in the nucleus and the breakdown of the nuclear membrane. Finally, with the breakdown of the plasma membrane, histones are released into the extracellular space (72, 73). As a proinflammatory DAMP, histones activate the downstream MyD88 and NF-κB signaling pathway by interacting with TLR2, TLR4, and TLR9, and produce inflammatory cytokines such as IL-6, TNF-α, and MCP1, exacerbating the renal inflammatory response (64, 74). NETs can also be actively secreted from living neutrophils through vesicular transport (71, 75).

### 2.3 Interleukin-1

Interleukin-1α (IL-1α) and interleukin-33 (IL-33) are members of the interleukin-1 (IL-1) family that are both cytokines and can act as DAMPs, which are passively released by necrotic cells into the extracellular space due to plasma membrane destruction (76–78). IL-1α can also be actively released by macrophages through gasdermin D (GSDMD) pore formation during pyroptosis, a form of regulated cell death (77, 79). IL-1α is present and biologically active in epithelial cells under physiological conditions (80). Upon external stimuli, IL-1α is released from damaged cells and binds to the IL-1 receptor (IL-1R) on nearby cells, activating the NF-κB signaling pathway, inducing TNF-α production, and mediating the recruitment of immune cells, which promotes kidney injury (34, 81–83). IL-33 is constitutively expressed in the nucleus of endothelial, epithelial, and other structural cells in the physiological state (84–86). IL-33 is a pleiotropic cytokine that mediates tissue inflammation and repair by binding to the specific receptor tumorigenicity 2 (ST2). ST2 is expressed on the surface of multiple immune cells, including nature killer (NK) cells, regulatory T cells (Tregs), neutrophils, macrophages, B cells, and natural killer T (NKT) cells (87). In mice model of IRI-induced AKI, IL-33 was passively released by dying endothelial cells and bound to ST2 as a ligand, which induced the conversion of proinflammatory M1 macrophages into anti-inflammatory M2 macrophages, reducing the release of proinflammatory factors, whereas IL-33 promotes the expansion of type II innate lymphoid cells (ILC2s) and Tregs, improving the immune microenvironment and reducing renal inflammation (88, 89). Interestingly, IL-33 released into the extracellular space can also act as a proinflammatory DAMP targeting invariant NKT (iNKT) cells, recruiting neutrophils to infiltrate the kidney and amplifying inflammatory injury in a mouse model of IRI-induced AKI (90–92). IL-33 exhibits dual functions in AKI, which may depend on the type of immune cells activated by IL-33 and the specific immune microenvironment, which will require more in-depth studies to elucidate the complex regulatory mechanisms of the IL-33/ST2 signaling pathway in AKI.

### 2.4 Uric acid

Uric acid is generated through the metabolic degradation of purines, approximately 70% of it is excreted via the kidney, and it has long been recognized as a significant risk factor for kidney-related diseases (93, 94). Uric acid is released passively from the damaged cells. Uric acid, particularly in its crystalline form, can act as a proinflammatory DAMP. Uric acid crystals bind to TLRs (e.g., TLR2 and TLR4), providing priming signals, and activate the NLRP3 inflammasome to induce the production of IL-1β, TNF-α, and MCP1, and induce the recruitment of immune cells, exacerbating renal inflammatory responses (28, 95–97). Many clinical approaches for the treatment of kidney-associated disorders, such as allopurinol and febuxostat administration, can effectively attenuate renal function partly by reducing circulating uric acid concentrations (98). This conclusion was validated in animal experiments, where allopurinol improved renal function by lowering the serum uric acid concentration in animal models of heat stress, rhabdomyolysis, and exercise-induced AKI (99, 100). Febuxostat can ameliorate contrast and IRI-induced AKI by the same mechanism (101–103). In-hospital mortality in patients with AKI appears to be associated with high serum uric acid levels, which is expected to be a prognostic marker for AKI (104).

### 2.5 Adenosine triphosphate

ATP serves as a vital energy source for cells, and mitochondrial oxidative phosphorylation regulates intracellular ATP levels (105, 106). ATP can be released into the extracellular space either passively or in a regulated manner. Passive release occurs from damaged or necrotic cells through plasma membrane rupture (22, 107). In contrast, regulated ATP release from living cells occurs via specific pathways such as pannexin or connexin hemichannels, as well as vesicular transport mechanisms (108, 109). Extracellular ATP is recognized as a proinflammatory DAMP that binds to the P2X7 purinergic receptor (P2X7R), causing potassium efflux, which activates NLRP3 inflammasome (110). In IRI and sepsis-induced AKI, ATP can activate NLRP3 inflammasome by binding to P2X7R, promoting the production of proinflammatory factors including IL-1β, IL-6, and MCP1 and impairing the kidney (111, 112). P2X7R knockdown ameliorated IRI and cisplatin-induced AKI and AKI progression to renal fibrosis by inhibiting the activation of NLRP3 inflammasome (112, 113). Similarly, studies have indicated that the use of P2X7R antagonists mediates the inactivation of NLRP3 inflammasome and treats IRI and sepsis-induced AKI (114, 115). This predicts an optimistic therapeutic prospect for P2X7R antagonists in AKI of multiple etiologies.

### 2.6 Mitochondrial DNA

mtDNA, a double-stranded circular DNA molecule, is involved in encoding the composition of the core subunits of the respiratory chain, which is important for the maintenance of mitochondrial function and cellular metabolism (116, 117). In recent years, mtDNA has also been recognized as a proinflammatory DAMP that plays a role in various diseases (118, 119). Depletion of mtDNA has been observed during AKI, which is closely related to the degree of renal damage (120). During cellular stress, mitochondrial homeostasis is disrupted, prompting the opening of the mitochondrial permeability transition pore, followed by the release of mtDNA into the cytoplasm, The leaking mtDNA acts as an endogenous ligand for the cyclic GMP–AMP synthase (cGAS), which triggers type I interferon responses via the stimulator of interferon gene (STING) signaling pathway, and also activates the classical NF-κB inflammatory response, which produces inflammatory cytokines such as TNF-α and IL-6 (118, 121). By binding to TLR9, mtDNA can also activate NF-κB transcription, leading to the release of proinflammatory factors and immune cell infiltration, amplifying the inflammatory response (122–124). In addition, mtDNA can directly activate NLRP3 inflammasome and enhances the release of the inflammatory cytokines IL-1β and IL-18 (125–127). Some studies have shown significant correlations among urinary mtDNA level and serum creatinine, eGFR, and the AKI marker neutrophil gelatinase-associated lipocalin (NGAL), suggesting the promising potential of urinary mtDNA as a biomarker for predicting AKI severity, which needs to be further validated by larger, multicenter-like cohort studies in the future to establish the role of urinary mtDNA in predicting AKI severity (128–130).

### 2.7 Uromodulin

Uromodulin, also known as Tamm-Horsfall protein (THP), is a kidney-specific glycoprotein produced mainly by the epithelial cells of the ascending limb of the Henle loop, which is excreted into the urine mainly by apical secretion, and is one of the most abundant urinary proteins (131). Physiologically, uromodulin is not immunologically active in the tubular lumen of the renal tubule but serves multiple protective functions, such as resisting urinary tract infections and preventing urinary stone formation (132–134). However, when renal tubular cells are damaged, uromodulin may be partially leaked into the renal interstitial compartment, and mislocalized uromodulin is thought to be a proinflammatory DAMP, which induces renal inflammation by binding to TLR4, activating NLRP3 inflammasome, and inducing the activated aggregation of immune cells and secretion of the proinflammatory factor IL-1β (135–137). The specific receptors and molecules involved in immune cells modulation by uromodulin leaking from the renal interstitial compartment require more in-depth studies.

### 2.8 Biglycan

Biglycan, a small leucine-rich proteoglycan expressed mainly in the renal mesenchyme and present in the extracellular matrix (ECM) in the physiological state, is a key ECM component that plays an important role in angiogenesis, inflammation, and autophagy (136, 138). Dying cells can release biglycan through protein hydrolysis. The released biglycan exists in its soluble form and is seen as a DAMP, which exerts proinflammatory function by binding to the corresponding receptors (19). Biglycan can activate the downstream MyD88 and NF-κB inflammatory signaling pathway by binding to TLR2 and TLR4, inducing the infiltration of various immune cells such as T-cells and neutrophils, polarization of M1 macrophages, and production of TNF-α, IL-1β, C-C motif chemokine ligand 2 (CCL2) and CCL5, thereby exacerbating the inflammatory injury in IRI-induced AKI (139–141).

### 2.9 Hyaluronic acid

HA is another important ECM component. It is primarily composed of repeating disaccharide chains of D-glucuronic acid and D-N-acetylglucosamine, which play important roles in maintaining tissue integrity and regulating cellular functions (142–144). The biological function of HA depends on its molecular weight, and in the physiological state, it exists mainly as high-molecular-weight HA (HMW-HA) (145, 146). HMW-HA binds to CD44, a main receptor of HA, to promote the polarization of M1 to M2 macrophages and inhibit NF-κB activation, reducing the expression of inflammatory factors including IL-6 and IL-8 and thus reducing systemic inflammation and maintaining immune balance (147–149). During cell injury, HMW-HA in the ECM is degraded by hyaluronidases to low-molecular-weight HA (LMW-HA), which is regarded as a DAMP. By binding to TLR2 and TLR4, LMW-HA activates dendritic cells and activates the NF-κB signaling pathway, increasing the release of the proinflammatory cytokines IL-1β, IL-6, TNF-α, and IL-12 (145, 150). LMW-HA also induces the expression of chemokines such as CCL3 and CCL4, recruiting immune cells and exacerbating inflammatory responses (150, 151). The inhibition of HMW-HA degradation alleviates renal inflammation and protects renal function in a mouse model of IRI-induced AKI (152).

### 2.10 S100A8/A9

S100A8 and S100A9 are calcium-binding proteins that belong to the S100 protein family and are usually present as heterodimers in a high-calcium environment. The S100A8/A9 heterodimer is predominantly expressed in the cytoplasm of myeloid cells and is involved in regulating the polymerization of microtubules (153). S100A8/A9 released into the extracellular space is regarded as a DAMP and helps in regulating immune responses (154, 155). S100A8/A9 can be actively secreted or passively released into the extracellular space. Passive release occurs through dying cells or GSDMD pore, or by neutrophils through the formation of NETs, whereas active secretion occurs mainly by macrophages via the lysosomal exocytosis pathway (153, 156, 157). In a mouse model of IRI-induced AKI, S100A8/A9 activates the NF-κB signaling pathway by binding to TLR4 and promotes the infiltration of the proinflammatory cytokines IL-6, IL-1β, and TNF-α and the aggregation of macrophages and neutrophils, thereby exacerbating renal inflammation (157–159). A study revealed that S100A8/A9 inhibitor attenuates the binding of S100A8/A9 to TLR4, alleviates the inflammatory response to sepsis-induced AKI, and protects the kidney (160).

## 3 Conclusions

Despite current advances in investigating the pathological mechanisms of AKI, the lack of diagnostic and prognostic biomarkers for AKI and specific AKI therapeutic strategies are major clinical challenges that need to be addressed. This review preliminarily explored the release mechanisms and functions of DAMPs in regulating immune responses, revealing that not all DAMPs are exacerbating factors for AKI; some DAMPs promote AKI repair and protect the kidney, which may depend in part on the specific immune microenvironment or molecular weight in which the DAMPs are located. In the mouse model of IRI-induced AKI, IL-33 binds to ST2 on the surface of macrophages, ILC2s, and Tregs, reducing renal inflammation; however, IL-33 also binds to ST2 on the surface of iNKT cells, recruiting neutrophils and amplifying inflammatory injury. The complex mechanisms by which the IL-33/ST2 signaling pathway expresses a dual function in AKI may be due to the binding of IL-33 to ST2 on the surface of different immune cells and the specific immune microenvironment in which it resides. The complex mechanisms through which the IL-33/ST2 signaling pathway expresses a dual function in AKI warrant more in-depth investigation. The dual function of HA depends on its molecular weight. In the physiological context, HA predominantly exists as HMW-HA, which can inhibit NF-κB activation, reduce the expression of inflammatory factors such as IL-6 and IL-8, and exhibit anti-inflammatory function. Conversely, during stress, HMW-HA is degraded by hyaluronidases to LMW-HA, which acts as a proinflammatory DAMP. Moreover, serum uric acid was highly correlated with in-hospital mortality in patients with AKI, and urinary mtDNA was significantly correlated with serum creatinine, eGFR, and NGAL, which all appear to indicate that some DAMPs have predictive value for AKI. In addition, antagonists or neutralizing antibodies targeting some of the DAMPs (HMGB1 and S100A8/A9) or PRR (P2X7R) could protect to a certain extent against multiple etiologically induced AKI, which in turn highlights the great potential of DAMPs in clinical therapy. However, further studies are needed, including the identification of more DAMPs and elucidation of the detailed pathway and relationship with clinical features, to help us identify AKI early and contribute to the development of drugs for the treatment of AKI.
